# Comparison of 2D 4K vs. 3D HD laparoscopic imaging systems in bariatric surgery: study protocol for a randomized controlled prospective trial

**DOI:** 10.1186/s13063-024-07983-4

**Published:** 2024-02-22

**Authors:** Tibor A. Zwimpfer, Nadja Stiegeler, Philip C. Müller, Andreas Schötzau, Bernhard Fellmann-Fischer, Viola Heinzelmann-Schwarz, Ralph Peterli, Marko Kraljević

**Affiliations:** 1 Department of Obstetrics and Gynecology, University Hospital Basel, Basel, 4056 Switzerland; 2 Medical Faculty, University Basel, Basel, 4056 Switzerland; 3 Department of Visceral Surgery, Clarunis - University Center for Gastrointestinal and Liver Diseases, St. Clara Hospital and University Hospital Basel, Basel, 4002 Switzerland

**Keywords:** Laparoscopy, 2D 4K vision system, 3D HD vision system, Bariatric surgery, Gastric bypass, Operating time, Workload, Surg-TLX

## Abstract

**Background:**

Vision is an important and defining element of laparoscopy and significantly affects the outcome of surgery in terms of time, error, and precision. Several new imaging systems have become available for laparoscopic surgery, including three-dimensional (3D) high-definition (HD) and two-dimensional (2D) ultra-high-resolution (4K) monitors. 3D HD systems offer a number of potential benefits to surgeons and patients over traditional 2D systems, including reduced operating time, blood loss, and hospital stay. However, the performance of 3D systems against the new, ultra-high definition 4K systems is barely known and highly controversial. There is a paucity of studies comparing them in clinical settings. The aim of this study is to compare 2D 4K and 3D HD perspectives in gastric bypass surgery.

**Methods:**

Forty-eight patients with an indication for gastric bypass will be randomized to receive laparoscopic gastric bypass surgery using either 2D 4K or 3D HD systems. The operations will be performed by a well-coordinated team of three senior surgeons. The primary outcome is operative time. Secondary outcomes include intraoperative complications, blood loss, operator workload as assessed by the validated Surg-TLX questionnaire, and postoperative complications according to the Clavien-Dindo classification. An interim analysis is planned after enrollment of 12 participants for each group.

**Discussion:**

This prospective, randomized trial is designed to test the hypothesis that the use of a 3D HD system will result in a significant improvement in operative time compared to a 2D 4K system in bariatric surgery. The objective is to provide clinical evidence for new laparoscopic imaging systems and to evaluate potential benefits.

**Trial registration:**

This trial is registered at clinicaltrials.gov under the identifier NCT05895058. Registered 30 May 2023. BASEC2023-D0014 [Registry ID Swissethics, approved 3 May 2023]. SNCTP000005489 [SNCTP study register, last updated 13 July 2023].

**Supplementary Information:**

The online version contains supplementary material available at 10.1186/s13063-024-07983-4.

## Administrative information

Note: the numbers in curly brackets in this protocol refer to SPIRIT checklist item numbers. The order of the items has been modified to group similar items (see http://www.equator-network.org/reporting-guidelines/spirit-2013-statement-defining-standard-protocol-items-for-clinical-trials/).

**Table:** 

Title {1}	Comparison of 2D 4K vs. 3D HD laparoscopic imaging systems in bariatric surgery: study protocol for a randomized controlled prospective trial
Trial registration {2a and 2b}.	This trial is registered at clinicaltrials.gov under the identifier NCT05895058. Registered 30 May 2023. BASEC2023-D0014 [Registry ID Swissethics] SNCTP000005489 [SNCTP study register]
Protocol version {3}	Version 2.0 of date 08/04/2023
Funding {4}	Internal funding/In-house resources. All data were collected as part of routine treatment or follow-up of a gastric bypass operation. The medical devices (MD) are provided by the University Hospital Basel.
Author details {5a}	Dr. med. Tibor Andrea Zwimpfer Department of Obstetrics and Gynecology Department of Biomedicine University Hospital Basel Spitalstrasse 21 4056 Basel, Switzerland tiborandrea.zwimpfer@usb.ch Nadja Stiegeler Medical Faculty University of Basel Klingelbergstrasse 61 4056 Basel, Switzerland nadja@stiegeler.ch PD Dr. med. Philip C. Müller Department of Visceral Surgery Clarunis - University Center for Gastrointestinal and Liver Diseases, St. Clara Hospital and University Hospital Basel 4002 Basel, Switzerland philip.mueller@clarunis.ch Andreas Schötzau Department of Obstetrics and Gynecology University Hospital Basel Spitalstrasse 21 4056 Basel, Switzerland andreas.schoetzau@usb.ch Dr. med. Bernhard Fellmann-Fischer Department of Obstetrics and Gynecology University Hospital Basel Spitalstrasse 21 4056 Basel, Switzerland bernhard.fellmann@usb.ch Prof. Dr. med. Viola Heinzelmann-Schwarz Department of Obstetrics and Gynecology Department of Biomedicine University Hospital Basel Spitalstrasse 21 4056 Basel, Switzerland viola.heinzelmann@usb.ch Prof. Dr. med. Ralph Peterli Department of Visceral Surgery Clarunis - University Center for Gastrointestinal and Liver Diseases, St. Clara Hospital and University Hospital Basel 4002 Basel, Switzerland ralph.peterli@clarunis.ch PD Dr. med. Marko Kraljević Department of Visceral Surgery Clarunis - University Center for Gastrointestinal and Liver Diseases, St. Clara Hospital and University Hospital Basel 4002 Basel, Switzerland marko.kraljevic@clarunis.ch
Name and contact information for the trial sponsor {5b}	PD Dr. med. Marko Kraljević Department of Visceral Surgery Clarunis – University Center for Gastrointestinal and Liver Diseases, St. Clara Hospital and University Hospital Basel 4002 Basel, Switzerland + 41 61,777 73 15 marko.kraljevic@clarunis.ch
Role of sponsor {5c}	Marko Kraljević is the Sponsor-Investigator and one of the operating surgeons in the study. Together with his surgical team, he will recruit patients, inform them about the study and obtain informed consent. He will be responsible for performing the surgeries and collecting the data. After surgery, he will provide the data to the other authors of the study for further processing, analysis, and interpretation, and will write the report. He has final authority over all activities.

## Introduction

### Background and rationale {6a}

Laparoscopy has become an essential part of modern surgery. However, there are still some limitations to this technique [1]. In particular, vision is an important and defining element of laparoscopy and significantly affects the outcome of surgery in terms of time, intraoperative errors, and surgical precision [2]. Since the introduction of laparoscopic surgery, this surgical practice has evolved rapidly and many technological advances have been made to overcome the shortcomings of early endovision systems [3]. These advances have focused on improving the quality of vision and compensating for the loss of binocular depth perception [4]. Particularly for novices, the reduction from real three-dimensional (3D) vision to virtual two-dimensional (2D) vision is challenging and associated with a slower learning curve. Recently, several new imaging technologies have become available for laparoscopic surgery, including stereoscopic 3D high-definition (HD) and 2D ultra-high-resolution (4K) monitors. These developments in high-definition and stereoscopic imaging have attempted to overcome the major technical limitations of laparoscopy [5–7].

In experimental and clinical settings, several studies have been published in recent years suggesting that 3D systems offer a number of potential benefits to surgeons and patients compared to conventional 2D systems [2, 5]. Compared to the 2D system, 3D systems increase the subjective impression of safety and efficiency and consequently provide better surgeon confidence during surgery due to the depth perception that enables better visualization [2, 8]. In addition, the 3D HD imaging system significantly reduces operating time and blood loss, as well as shortens hospital stays [2]. This is critical as prolonged operative time and immobilization are known to be independent predictors of post-operative complications [9]. In 2018, the European Association for Endoscopic Surgery (EAES) published recommendations that 3D systems should be used in clinical practice to reduce operative time [2]. Despite these recommendations and the advantages of 3D imaging, the system is not widely used due to the need to train surgical staff in the technology and modernize operating rooms, both of which are associated with higher costs [10]. In addition, the potential superiority of 3D systems over new ultra-high-definition (4K) systems remains unproven and therefore highly controversial.

Indeed, with the introduction of 4K monitors, 2D rendering can provide four times the visual quality of conventional HD. This system improves vision through ultra-high resolution, a wider range of colors, and enhanced visualization, potentially providing a more realistic image and stronger monocular depth perception. The improved visualization allows surgeons to see tissue structures up close and evaluate them more accurately. Both novice and experienced surgeons have been shown to have better outcomes in terms of operating time and errors when using the 2D 4K system compared to the 2D HD system [11]. Currently, the use of the new 2D 4K technology is expanding and may become the standard in the near future [12]. However, the major limitation of 2D laparoscopy is still the lack of depth perception. The surgeon must compensate by using secondary visual reference points to correctly interpret the 2D laparoscopic image. This disadvantage can potentially increase the surgeon’s workload, risk of error, and operating time.

The choice of laparoscopic tools should be based on the best available evidence, but there is a paucity of studies comparing 3D HD and 2D 4K systems in the clinical setting. Particularly for more complex procedures, there are few, although some studies have shown that the more complex the laparoscopic procedure involving multiplane interactions, the more surgeons benefit from 3D visualization [12–15]. Additionally, Dunstan et al. [13] compared 3D with 4K laparoscopy in performing cholecystectomies, while Kanaji et al. [14] performed a comparison in performing laparoscopic gastrectomy for gastric cancer. The conclusion of both studies was that the 3D HD laparoscopic system did not reduce operative time and provided similar clinical outcomes compared to 2D 4K.

In summary, the benefits of imaging systems can only be truly addressed in clinical trials with appropriately powered sample sizes. This prospective randomized controlled trial comparing 3D HD and 2D 4K perspectives in gastric bypass surgery was designed for this purpose.

### Objectives {7}

We aim to investigate the hypothesis that the use of the 3D HD system will result in a significant improvement in operative time compared to a 2D 4K system in bariatric surgery. This hypothesis is based on our recently published randomized controlled preclinical study comparing 2D 4K and 3D HD using tasks performed on a pelvitrainer model by experts and medical students. Participants in both groups, experts and medical students, achieved significantly better results, including faster task completion times and fewer errors, with the 3D HD imaging system compared to the 2D 4K system [8]. Based on these preclinical results, we will now conduct the randomized trial in a clinical setting. Due to the standardized surgical procedure and the comparable conditions of gastric bypass surgery, we expect to explore significant differences. In addition, to provide a comprehensive overview of the comparison of 2D 4K and 3D HD laparoscopy in a clinical setting, we will evaluate surgeon workload and intraoperative and postoperative complications, including hospital length of stay.

### Trial design {8}

This study is designed as a single-center, parallel-group, superiority, randomized, controlled, prospective trial. All patients will have gastric bypass surgery and will be operated with 3D HD or 2D 4K imaging depending on the intervention group assignment. The patient allocation is 1:1.

## Methods: participants, interventions, and outcomes

### Study setting {9}

This study is a single-center study and will be conducted at Clarunis, University Center for Gastrointestinal and Liver Diseases (St. Clara Hospital and University Hospital Basel), which is a certified reference center for bariatric and metabolic surgery with a caseload of nearly 80 complex bariatric surgeries per year. The surgeries are performed by three experienced senior bariatric surgeons from Clarunis at the University Hospital Basel.

### Eligibility criteria {10}

#### Inclusion criteria

Patients must meet the following criteria to be eligible for the study:

- Patients with an indication for gastric bypass according to the Swiss Morbid Obesity Group (SMOB) guidelines: age ≥ 18 years, BMI ≥ 35, and a cumulative 2 years of controlled conservative diet without weight loss
- Signed informed consent

#### Exclusion criteria

Patients who meet any of the following criteria at the screening visit are not eligible for the study:

- Patients who are ineligible for gastric bypass based on one or more of the following criteria: BMI ≥ 50, lack of adequate weight loss therapy for 2 years, malignant disease, Child A cirrhosis, Crohn’s disease, carcinoma patients, severe mental illness requiring treatment (not due to obesity that has led to more than one inpatient psychiatric hospitalization in the last 2 years), chronic substance abuse, lack of compliance (missed appointments, inability to cooperate), lack of understanding of the requirements and conditions of postoperative therapy and treatment (confirmed by the specialist)
- Non-signed informed consent

### Who will take informed consent? {26a}

Patients evaluated for gastric bypass according to the SMOB guidelines at the Clarunis multidisciplinary obesity conferences are recruited into the clinical trial by the operating surgeon. The surgeon will inform the patient of the clinical trial and obtain informed consent. If the patient agrees to participate in the study and signs the informed consent, he or she will be enrolled in the study.

### Additional consent provisions for collection and use of participant data and biological specimens {26b}

This is not applicable; there is no collection and use of participant data or biological specimens in ancillary studies.

## Interventions

### Explanation for the choice of comparator {6b}

The comparison group to 2D 4K imaging for performing laparoscopic gastric bypass is 3D HD imaging. The EAES has recently made recommendations that 3D systems should be used in the clinical setting to reduce operative times [2]. However, its use is still limited due to higher costs and lack of familiarity. Other weaknesses of 3D imaging to date have been the need to wear special glasses and the reported side effects for surgeons associated with 3D vision systems such as dizziness, eye fatigue, nausea, and headaches [11]. However, rapid advances in 3D imaging technology over the past two decades appear to have successfully overcome previous barriers to widespread clinical use. Current 3D systems capture separate images using either two separate rod lenses or two separate chips on a scope tip to provide two vertically separated images that simulate binocular vision [16]. These improvements have made 3D imaging more user-friendly, with fewer side effects, and its benefits are becoming more apparent.

### Intervention description {11a}

The two medical devices to be compared have components that are as similar as possible and are used under the same conditions. The brand of both laparoscopic towers and all listed components are from Karl Storz SE & Co., Tuttlingen, Germany. The two camera systems used are a 2D 4K system module (TC304 IMAGE1 STM 4 U-LINK) equipped with a 30° field of view and a 10-mm diameter laparoscope (HOPKINS Optic) and a 3D HD system module (TC300 IMAGE1 STM D3-LINK and TC302 IMAGE1 STM H3-LINK) equipped with a 30° field of view and a 10.3-mm diameter laparoscope (TIPCAM1 SPIES 3D LAP Optic). The 4K endoscopy tower is equipped with a 32″ 4K monitor (TM 343) with a screen resolution of 3840 × 2160, while the 3D endoscopy tower is connected to a 32″ 3D monitor (TM 323) with a corresponding screen resolution of 1920 × 1080. 3D glasses, also from Karl Storz SE & Co., Tuttlingen, Germany, are used for the 3D system. The surgeons are positioned exactly one and a half meters in front of the monitor to ensure the effect of both the 4K and 3D imaging systems.

The comparison of the two laparoscopic imaging systems 2D 4K and 3D HD is performed during a gastric bypass surgery. This procedure provides a high level of comparability as it is highly standardized, complex, and involves many multiplane interactions. These gastric bypass operations are all performed by three defined senior surgeons of the Clarunis bariatric surgery team as described in detail above.

### Surgery procedure

**Fig. 1:**
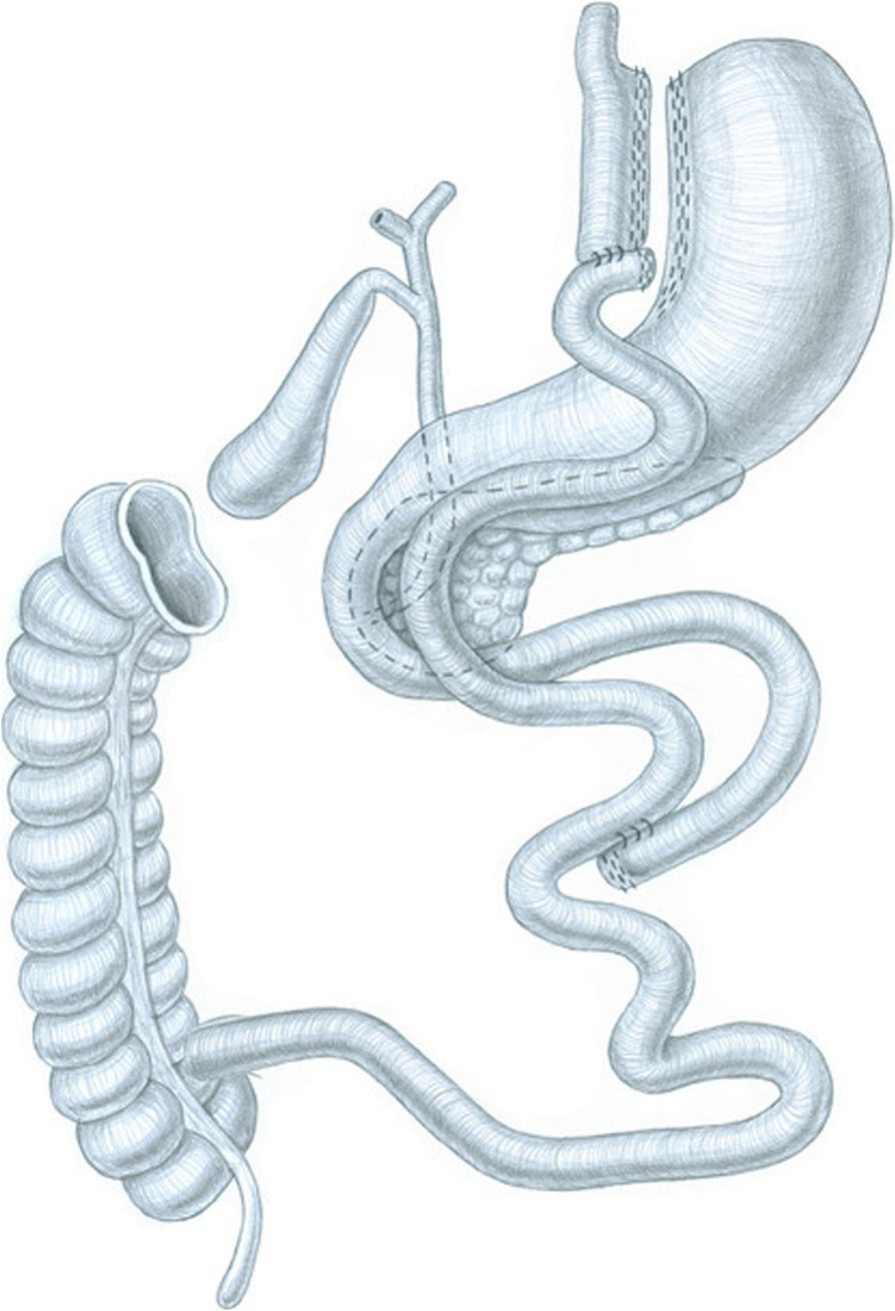
Visualization of the gastric bypass operation

All patients will receive a laparoscopic Roux-en-Y gastric bypass (LRYGB), an effective surgical treatment for obesity (Fig. 1). Briefly, the upper part of the stomach is separated from the rest of the stomach below the esophagus by stapling sutures, creating a gastric pouch. The upper part of the small intestine is cut and one limb is attached to the stomach pouch. The small intestine, which comes from the blindly closed stomach and contains the digestive juices from the duodenum, is sewn into this raised loop of small intestine. This bypasses the rest of the stomach (= bypass) [17].

### Criteria for discontinuing or modifying allocated interventions {11b}

Gastric bypass surgery and its pre- and post-operative procedures are performed as standard. The study only influences the choice of laparoscopic imaging used during surgery. Consequently, the criteria for stopping or modifying the allocated procedures for a given subject will depend on the responsible operator in consultation with their multidisciplinary obesity team and within the given national guidelines of the SMOB. If the indication is no longer given due to changed inclusion or exclusion criteria, the intervention will not be performed. In addition, patients may withdraw from the study at any time for any reason without consequence.

Patients excluded prior to gastric bypass surgery will be replaced to maintain the required number of procedures for comparison. Patients who are excluded after surgery because they do not return for their first post-operative visit at 3 months, and therefore no post-operative follow-up can be documented, will not be replaced, as this follow-up is only one of the secondary endpoints of the study.

### Strategies to improve adherence to interventions {11c}

This is not applicable, as the operations are performed according to the standard for gastric bypass surgery and are therefore already subject to its strict regulations. This study only defines the laparoscopic imaging used. Proper planning and execution is the responsibility of the operating surgeon. Monitoring is defined in a separate monitoring plan according to the ISO 14155 guidelines.

### Relevant concomitant care permitted or prohibited during the trial {11d}

This is not applicable; no relevant concomitant care and interventions are allowed or prohibited during the study. If unexpected events occur during surgery that require additional interventions, this will be documented in detail and mentioned and considered in the analysis.

### Provisions for post-trial care {30}

This is not applicable as both medical devices (3D HD and 2D 4K) are already routinely used in bariatric surgery. Therefore, there are no additional risks associated with the conduct of the study, and no consequences are expected for patients. In case of problems or complications, the patient will be followed up as part of the post-operative procedure of a general gastric bypass surgery.

### Outcomes {12}

#### Primary

The primary outcome of our prospective study is operating time. It is the most important and decisive parameter in our comparison between 3D HD and 2D 4K and potentially influences our secondary outcomes. In summary, operative time has a significant impact on a patient’s postoperative outcome and is therefore of considerable clinical relevance. Operative time is measured and documented during the performance of laparoscopic gastric bypass. It is defined as the time from the beginning of the operation by the incision of the skin to the end of the operation by the end of the skin suture. The operative time is the difference between these two times and is documented in minutes.

#### Secondary

Secondary outcomes include intraoperative complications, operator workload, and postoperative complications. Intraoperative complications are assessed descriptively by the surgeon’s documentation in the operative report. These include major bleeding, injuries or burns to surrounding organs (e.g., stomach, esophagus, spleen, liver), circulatory problems, or unexpected intra-abdominal findings that deviate from the standardized surgical procedure or expected extent and must be noted in the operative report. Blood loss is recorded and documented quantitatively in milliliters. In addition, a postoperative Hb control is performed if indicated by intraoperative complications and the Hb decrease is determined. Immediately after each surgery, the surgeon completes the Surg-TLX questionnaire, a validated multidimensional measure of surgical workload. The Surg-TLX defines workload based on six dimensions: mental demands, physical demands, temporal demands, task complexity, situational stress, and distractions. The condition of the operating surgeon has a great influence on the outcome of an operation. Especially when using 3D imaging, some undesirable side effects have been reported. It is important to determine the subjective workload of the surgeon when using modern imaging and performing a more complex procedure. Postoperative complications are influenced by many factors, including operative time and intraoperative complications. It is important to capture these in order to compare the postoperative outcomes of the two imaging systems. Postoperative complications are classified and documented up to 90 days according to the internationally accepted Clavien-Dindo classification. In addition, the length of hospital stay is evaluated. The standard length of hospital stay is three nights or 4 days, defined from the day of surgery, which is also the day of admission, up to and including the day of discharge. Other outcomes of interest are confounding parameters that we will consider in the statistical analysis. These include age, sex, and BMI of the operated patients. Another possible parameter, if available, is the number of previous abdominal surgeries a patient has had. Previous abdominal surgeries, especially upper abdominal surgeries, may be a complicating factor and will be reported and considered accordingly. Furthermore, it is documented exactly which operation was performed by which of the three surgeons as well as their personal prevalence (2D 4K vs. 3D HD). This is to analyze a possible bias.

### Participant timeline {13}

**Fig. 2:**
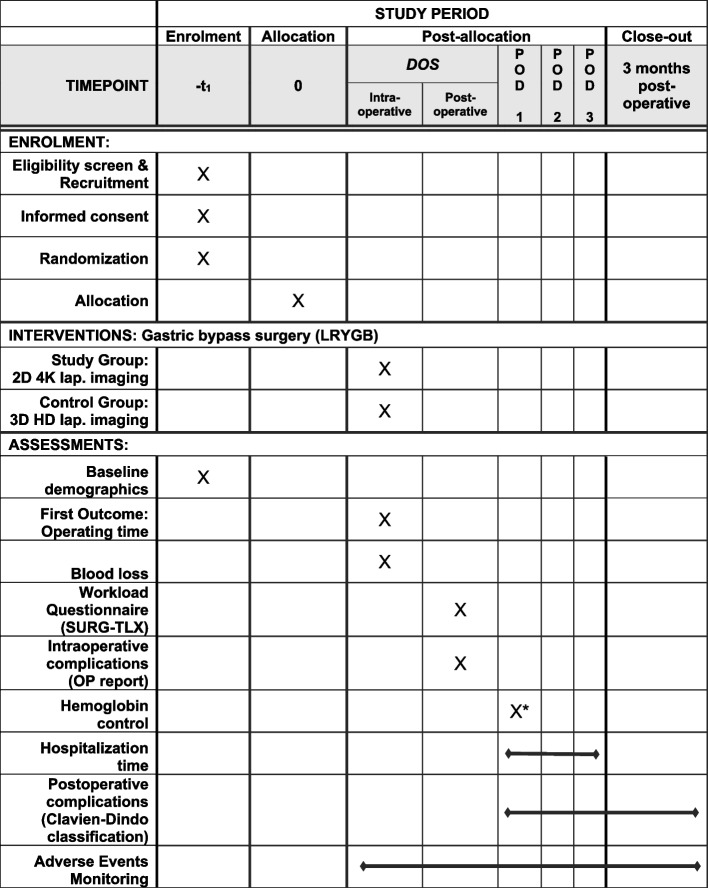
SPIRIT figure: Schedule of enrollment, interventions, and assessments. Asterisk symbol (*) indicates the following: if indicated in case of intraoperative complications. DOS, day of surgery; POD, postoperative day; LRYGB, laparoscopic Roux-en-Y gastric bypass; SURG-TLX, surgery task load index

The participant timeline is presented in the SPIRIT figure: schedule of enrollment, interventions, and assessments (Fig. 2).

### Sample size {14}

The sample size was calculated for the primary outcome (operating time). As the study is mainly based on knowledge from preclinical studies and there is not much guiding literature in the clinic, some assumptions had to be made at the beginning. We assume that an operator needs approximately 75 min to perform a gastric bypass using 3D HD imaging, while performing the exact same operation using 2D 4K imaging takes 90 min. This assumption is supported by the literature, which states that a gastric bypass operation takes approximately 90 min for experienced operators using 2D laparoscopy, by the results of studies that showed a time savings of approximately 15 min using 3D laparoscopy, and by the results of our preliminary study, which showed an average time savings of almost one fifth using 3D laparoscopy compared to 2D 4K [18–20]. The EAES systematic review also suggests an average difference of 15 min for procedures involving laparoscopic suturing [2]. Calculation is as follows: sequential analysis with a maximum of 2 looks (group sequential design, O’Brian-Fleming alpha spending), overall significance level 2.5% (one-sided); the sample size was calculated for a two-sample *t*-test, minimal detectable difference = 15, standard deviation = 15, power 90%; futility bound of 0 at interim analysis; number of subjects at interim 22.3, final: 44.6. Sample size calculation is based on RPACT (R package version 3.2.1) [21].

Therefore, we will recruit 48 patients in blocks of four with gastric bypass indications and randomize them to the two groups (2D 4K and 3D HD).

### Recruitment {15}

The surgeon recruits patients for the study. These are patients who have already decided to undergo gastric bypass surgery independently of the study and who meet all of the indications for the procedure. Recruitment for the study is therefore dependent on the general performance of gastric bypass surgery at Clarunis and will continue until the defined sample size is reached. Given that Clarunis is a certified reference center for bariatric surgery and that gastric bypass is the most common type of bariatric surgery, we expect the study to take approximately 2 years to complete.

## Assignment of interventions: allocation

### Sequence generation {16a}

Patients are randomly assigned to the four possible sequences using block randomization with a block size of four patients. The two groups will be assigned according to the randomization list, and surgery will be performed based on these groups using either 2D 4K or 3D HD imaging. If a patient fails to complete the study, the next patients will follow the list at the same point as the failed patient. There is no prior stratification.

### Concealment mechanism {16b}

This is not applicable as the study is single-blind. It is only the patient who is not informed of the imaging technique used; the surgeon is informed.

### Implementation {16c}

Patients are randomized 1 day before surgery using a computer-generated allocation list (generated using R version 4.1.3). The randomization list is linked to the study’s data management program (REDCap) by the project statistician. The operating surgeon recruits the patients and collects the data needed to conduct the study. The day before surgery, the surgeon enters the data into the electronic case report form (eCRF) in REDCap, which is designed for this study. Once the patient is enrolled in the study and the required data is entered into the system, the patient is automatically assigned to an intervention group using the linked randomization list. This ensures that surgeons have no influence on patient allocation. However, they are unblinded and are informed on the preoperative day which laparoscopic system will be used for the next surgery in the study. This is unavoidable, which is why our study is single-blind. Only the patients are blinded.

## Assignment of interventions: blinding

### Who will be blinded {17a}

The study is single-blind. The patients are blinded, and they do not know which group they belong to, while the investigators are not blinded. The blinding of the patients is ensured by the fact that there is no visible difference between the two groups, as well as by standardized questions and discussion patterns of the investigators, so that no relevant information is revealed. Whether the laparoscopic surgery is performed with 2D 4K or 3D HD imaging is irrelevant for the preoperative assessment and the subsequent procedure. This also applies to the follow-up procedure.

### Procedure for unblinding if needed {17b}

This is not applicable; there is no situation that would require unblinding the patient.

## Data collection and management

### Plans for assessment and collection of outcomes {18a}

Data will be derived from electronic patient records and collected with an electronic case report form (eCRF) using REDCap electronic data capture tools hosted at the University of Basel. REDCap (Research Electronic Data Capture) is a secure, web-based software platform designed to support data collection for research studies [22, 23]. Laboratory testing is a standard part of the gastric bypass procedure and is performed in the central diagnostic laboratory of the University Hospital of Basel. The operative reports and other relevant information will be collected from the electronic patient records in the internal system of the University Hospital of Basel, and the information relevant to the study will be extracted and collected in the eCRF and in a folder created for the study. Only the study team has access to this specific study folder. The SURG-TLX is used to measure the surgeon’s workload immediately after surgery. The Surgery Task Load Index (SURG-TLX) is a validated multidimensional workload measure. It is a surgery-specific adaptation of the well-validated NASA-TLX and was proposed by Wilson et al. to assess the sources of workload perceived by surgeons in the operating room environment [24]. It has been used in robotic surgery in clinical settings and has been suggested to be useful in assessing human-machine interface issues and sources of stress caused by new technology in surgical practice. The questionnaire assesses operator workload along six dimensions:

- Mental demands: How mentally fatiguing was the procedure?
- Physical demands: How physically fatiguing was the procedure?
- Temporal demands: How hurried or rushed was the pace of the procedure?
- Task complexity: How complex was the procedure?
- Situational stress: How anxious did you feel while performing the procedure?
- Distractions: How distracting was the operating environment?

Postoperative complications are documented until 90 days postoperatively. Postoperative complications are classified into grades I–V according to the widely used and internationally accepted Clavien-Dindo classification [25].

### Plans to promote participant retention and complete follow-up {18b}

This is not applicable because the procedure and study follow-up (up to 90 days post-operatively) are performed as part of the standard gastric bypass procedure. In order to receive the gastric bypass indication, patients must sign a written consent for lifelong follow-up in the bariatric network at a certified center. This is in accordance with standard guidelines.

### Data management {19}

All source data are available at the University Hospital Basel. The source data include the original documents related to the study (signed informed consent, randomization codes) and the patient information contained in the electronic system of the University Hospital Basel. This includes the patient’s general data, medical history, and collected medical parameters (laboratory) as well as the operative reports and documented progress notes. All this data is stored in the internal system, to which authorized personnel have access. Data relevant to the primary and secondary outcomes are selectively transferred into REDCap by authorized personnel and collected in a GCP-compliant eCRF. The system allows for minimizing data entry errors through the development of branching logic and online data checks (e.g., range checks). Relevant documents such as the signed informed consent and operative report are also stored there. The operator workload questionnaire is completed directly online in the eCRF. In addition, (S)AEs are recorded in the eCRF.

Data will be exported by REDCap in CSV format and evaluated by the project statistician at interim and final analysis. The entire database will be exported at the end of the study and stored in the archives of the University Hospital Basel for a minimum of 10 years after the study has been terminated.

### Confidentiality {27}

Data entry and analysis will be done with anonymized data. Research data will be stored in the eCRF using a study identification code for each participant. The key to the list of identification codes will only be available to the research team during the study. Patient identification details will not be reported in publications.

### Plans for collection, laboratory evaluation, and storage of biological specimens for genetic or molecular analysis in this trial/future use {33}

This is not applicable; there was no collection of biological specimens.

## Statistical methods

### Statistical methods for primary and secondary outcomes {20a}

Data will be analyzed using rpact (R package version 3.2.1) [21]. Descriptive statistics of primary and secondary endpoints will include minimum, maximum, standard deviation, mean, median, interquartile, 25th percentile, and 75th percentile for each study group.

The primary endpoint (mean OT) will be compared between study groups using an independent *t*-test. Since the distribution of OT is expected to be approximately symmetric with no outliers, the *t*-test is the primary choice. The tests will be one-tailed at a 2.5% significance level. Based on the O’Brian-Fleming alpha power function, the critical *p*-value is 0.0015 at the interim analysis and 0.0245 at the final analysis. Stop for futility is non-binding and set > 0. Acceptance of H1 will be concluded if alpha < critical values at the appropriate stages.

Study parameters other than OT are considered secondary. Secondary endpoints will be compared exploratorily using Mann-Whitney *U*-tests or Fisher’s exact tests, as appropriate. Tests will be two-tailed, alpha = 0.05.

Study groups will be exploratorily adjusted for age, sex, BMI, and possibly other parameters using regression analysis.

### Interim analyses {21b}

An interim analysis is planned after enrollment of 12 participants for each group. Results and implications will be discussed with the project statistician, sponsor-investigator, and medical expert. An alpha power function based on O’Brian-Fleming boundaries will be used for alpha allocation at interim and final analysis. Details of the statistical significance cut-off are provided in the “Statistical methods for primary and secondary outcomes {20a}” section. In addition, a non-binding futility boundary of zero effect will be selected.

### Methods for additional analyses (e.g., subgroup analyses) {20b}

There are no subgroup analyses planned.

### Methods in analysis to handle protocol non-adherence and any statistical methods to handle missing data {20c}

To ensure data quality, the monitoring plan checks the data for missing data, extreme outliers, and erroneous entries prior to statistical analysis. Missing data will be closely monitored. However, missing data due to noncompliance or dropout should remain within the expected minimum. Dropouts related to the primary outcome will be replaced to achieve the required number of participants and thus significant results. There is no need to replace dropouts for the secondary outcomes, and there are no plans to use multiple imputation.

### Plans to give access to the full protocol, participant-level data, and statistical code {31c}

The datasets used and/or analyzed in the current study can be made available by the corresponding author upon reasonable request and in accordance with the research collaboration and data transfer guidelines of the University Hospital Basel.

## Oversight and monitoring

### Composition of the coordinating center and trial steering committee {5d}

This is a monocentric study designed, conducted, and coordinated at the University Hospital of Basel. The day-to-day management of the study is carried out by the following:

- Sponsor-investigator: takes over the supervision of the study and the medical responsibility for the patients, in addition he is one of the three defined operators of the study
- Medical expert: advises, conducted the corresponding preclinical study comparing 2D 4K vs. 3D HD on a pelvitrainer model [8]
- Study coordinator: enrollment, coordination of study visits, annual safety reports
- Statistician: organizes data collection, ensures quality, and analyzes data
- Other investigators/operators: recruit, obtain consent, operate, and ensure follow-up according to protocol

The study team meets as needed, but not regularly. There is no study steering committee or stakeholder/public engagement group.

### Composition of the data monitoring committee, its role and reporting structure {21a}

No specific DSMCs are required for this category A1 study. All interventions performed and the product tested are associated with extremely low risk factors, and therefore, any potential safety issues can and will be reported and handled in plenary by the sponsor-investigator and responsible personnel.

### Adverse event reporting and harms {22}

All adverse events (AEs), device defects (DDs), and serious adverse device effects (SADEs) reported by subjects or observed by investigators will be collected, fully investigated, and documented in the source document and appropriate case report form (eCRF; symptoms/diagnosis, event onset and cessation, event treatment and resolution, assessment of relationship to MD and/or investigational procedure) throughout the trial period. An annual safety report (ASR) will be submitted by the sponsor-investigator to the competent ethics committee (CEC) on an annual basis.

### Frequency and plans for auditing trial conduct {23}

Monitoring will be performed by a monitor (CRA) independent of the study team in accordance with ISO 14155. Based on the risk-based monitoring (RBM) score calculator of the Swiss Clinical Trial Organization (SCTO) monitoring platform, the estimated risk for this study is considered low. Based on this, monitoring will be limited to random visits (on-site and remote), including verification of the presence and completeness of the study file and CDM by the monitor, and will be the responsibility of the sponsor-investigator. CDM (with full SDV of key data) will be performed each time new subjects are enrolled or at least every 3 months. Data will be validated by persons authorized by the sponsor-investigator and under the supervision of the monitor prior to interim analysis (after *n* = 12 per group) and prior to final analysis. The monitoring plan can be consulted for further information. Possible inspections by health authorities may take place, where the source data/documents will be accessible to the inspectors and questions will be answered.

### Plans for communicating important protocol amendments to relevant parties (e.g., trial participants, ethical committees) {25}

The trial will be conducted in accordance with the protocol and the tenets of the Declaration of Helsinki in its current version, the European Medical Device Directive 2017/745 (MDD), ISO 14155 and ISO 14971 standards, ICH Good Clinical Practice (GCP) guidelines, as applicable, the Swiss Human Research Act (HRA) and its ordinances, and the requirements of the Swiss regulatory authority. No changes will be made to the protocol without prior approval of the sponsor-investigator and the Ethics Committee of Northwestern and Central Switzerland (EKNZ), except when necessary to eliminate obvious immediate hazards to subjects. All substantial amendments will be submitted to the EKNZ. Non-substantial changes are recorded and filed. They will be submitted to the EKNZ together with the annual safety report (ASR). If the changes concern or affect the participants in any way, they will be informed of the changes. If necessary, the registration in the SNCTP and in the primary WHO registry (ClinicalTrials.gov ) will be adjusted.

### Dissemination plans {31a}

International peer-reviewed journals will be used to fully disclose the results of this research. Both positive and negative results will be reported. Upon request, participants will receive a summary of the overall results of the study.

## Discussion

This randomized controlled prospective study is designed to compare 2D 4K vs. 3D HD laparoscopic imaging during gastric bypass surgery. The aim is to test the hypothesis that 3D HD laparoscopy can lead to significantly shorter operative times compared to the use of newer, high-quality 2D 4K laparoscopic systems in bariatric surgery. The results will be used to gather evidence on the imaging of modern laparoscopy. This evidence can then be used to focus on the advantages of modern imaging systems, to use them in a more targeted way, and to achieve better outcomes in laparoscopic surgery.

## Limitations

There are some limitations to be considered. First of all, it should be mentioned that, in daily practice, the laparoscopic imaging system is chosen according to the surgeon’s preference and the availability of medical equipment. As a result, the surgeon’s skills may adapt to the more common laparoscopic conditions. This represents a potential bias for direct comparison. To minimize this, we selected a team of three senior surgeons with extensive experience with both laparoscopic imaging systems. It is also important to note that there are many possible influences on operative time and secondary outcomes that are independent of the chosen laparoscopic imaging system. Due to the strict indication criteria for gastric bypass, the patients in the study should have similar conditions and therefore provide similar operative circumstances as much as possible. Comparability is further supported by the highly standardized nature of the gastric bypass procedure. In addition, it is important to mention that there is a general limitation of 4K technology due to the requirement of large size monitors (e.g., 55″) to benefit from the ultra-high resolution. When this is not the case, as in our randomized controlled trial or in the reality of many operating rooms, the surgeon should be close to the monitors to take full advantage of the 4K resolution. Otherwise, the 4K technology cannot be assumed to have an advantage.

## Strengths

This clinical trial will compare the two newest laparoscopic imaging systems available. As there is currently little relevant clinical literature on such a comparison in bariatric surgery, there are no significant disadvantages or even risks for patients participating in this study, regardless of which group they belong to. Both groups receive gastric bypass according to current standards. In contrast, further evidence on the two laparoscopic systems should provide some clinical advantages. As laparoscopic surgery continues to grow in popularity, clear evidence for the targeted use of laparoscopic imaging systems may further enhance its benefits. If 3D HD laparoscopy effectively leads to shorter operating times compared to 2D 4K systems, its targeted use in multiplane procedures such as gastric bypass may lead to better surgical outcomes for the patient. This in turn may have cost savings implications that may be of greater importance in the future as laparoscopic surgery gains momentum.

## Trial status

The current protocol is Version 2.0 of 08/04/2023. Recruitment started in August 2023. Patient recruitment is estimated to be completed June 2025.

### Supplementary Information

**Supplementary Material 1.**

## Abbreviations

AE: Adverse event
ASR: Annual safety report
CDM: Central data monitoring
CEC: Competent ethics committee
CRA: Clinical research associate
eCRF: Electronic case report form
2D: Two-dimensionality
3D: Three-dimensionality
DD: Device deficiency
DSMC: Data safety monitoring committee
EAES: Association for Endoscopic Surgery
GCP: Good Clinical Practice
H0: Null hypothesis
H1: Alternative hypothesis
HRA: Federal Act on Research involving Human Beings
ISO: International Organization for Standardization
MD: Medical device
MDR: Medical Device Regulation (EU) 2017/745 of 5 April 2017
LRYGB: Laparoscopic Roux-en-Y gastric bypass
OT: Operating time
SADE: Serious adverse device effect
SDV: Source data verification
SMOB: Swiss Morbid Obesity Group
SNCTP: Swiss National Clinical Trials Portal
