# Supplementary figures and images for: Comparison of 2D 4K vs. 3D HD laparoscopic imaging systems in bariatric surgery: study protocol for a randomized controlled prospective trial
Source: Trials. 2024 Feb 22;25:140. doi: 10.1186/s13063-024-07983-4 (PMC10885491; doi:10.1186/s13063-024-07983-4)

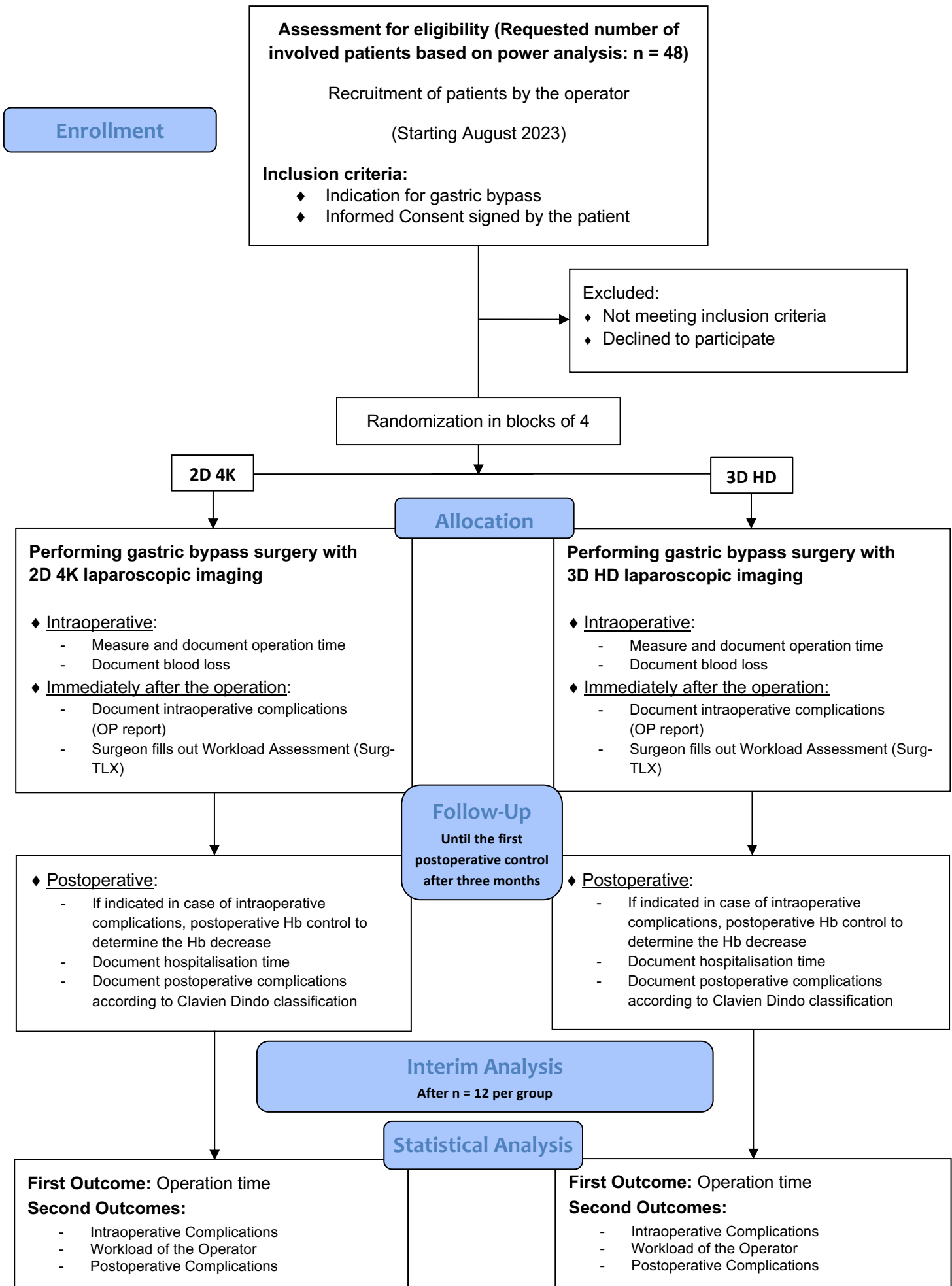

Supplement: Supplementary file 1 — Supplementary Material 1. [file 13063_2024_7983_MOESM1_ESM.pdf]
